# Bubble nucleation and growth on microstructured surfaces under microgravity

**DOI:** 10.1038/s41526-024-00352-0

**Published:** 2024-01-30

**Authors:** Qiushi Zhang, Dongchuan Mo, Seunghyun Moon, Jiya Janowitz, Dan Ringle, David Mays, Andrew Diddle, Jason Rexroat, Eungkyu Lee, Tengfei Luo

**Affiliations:** 1 Department of Aerospace and Mechanical Engineering, University of Notre Dame, Notre Dame, IN USA; 2 Space Tango, 611 Winchester Rd, Lexington, KY USA; 3 Department of Chemical and Biomolecular Engineering, University of Notre Dame, Notre Dame, IN USA

**Keywords:** Fluid dynamics, Porous materials

## Abstract

Understanding the dynamics of surface bubble formation and growth on heated surfaces holds significant implications for diverse modern technologies. While such investigations are traditionally confined to terrestrial conditions, the expansion of space exploration and economy necessitates insights into thermal bubble phenomena in microgravity. In this work, we conduct experiments in the International Space Station to study surface bubble nucleation and growth in a microgravity environment and compare the results to those on Earth. Our findings reveal significantly accelerated bubble nucleation and growth rates, outpacing the terrestrial rates by up to ~30 times. Our thermofluidic simulations confirm the role of gravity-induced thermal convective flow, which dissipates heat from the substrate surface and thus influences bubble nucleation. In microgravity, the influence of thermal convective flow diminishes, resulting in localized heat at the substrate surface, which leads to faster temperature rise. This unique condition enables quicker bubble nucleation and growth. Moreover, we highlight the influence of surface microstructure geometries on bubble nucleation. Acting as heat-transfer fins, the geometries of the microstructures influence heat transfer from the substrate to the water. Finer microstructures, which have larger specific surface areas, enhance surface-to-liquid heat transfer and thus reduce the rate of surface temperature rise, leading to slower bubble nucleation. Our experimental and simulation results provide insights into thermal bubble dynamics in microgravity, which may help design thermal management solutions and develop bubble-based sensing technologies.

## Introduction

Since the first expression of inertially controlled growth and collapse of vapor bubbles was developed by Lord Rayleigh in 1917^1^, the dynamics of surface bubbles have been extensively studied both experimentally and theoretically^2–10^. Understanding the dynamics of surface bubble nucleation and growth can help to formulate heat transfer models in a wide range of modern technologies, such as the cooling of electronics, refrigeration cycles, nuclear reactors, and metal industries, etc^11^. In addition to the traditional pool boiling, surface bubbles can also be generated through the photo-thermal evaporation process driven by enhanced surface plasmon resonance heating effect, and the corresponding mechanisms and applications (e.g., particle deposition and sensing) have been investigated in recent decades^12–18^.

Although extensive research has been done to study the dynamics of surface bubble nucleation and growth in various conditions and settings, most of these works were conducted in the terrestrial gravity environment. As we know, surface bubble nucleation and growth are initiated and dictated by the heat transfer between the heating surface and surrounding liquid, and the temperature of the heating surface can be significantly influenced by the liquid flow close to the bubble nucleation site^19–21^. Therefore, the bubble dynamics in the microgravity environment, i.e., in space, can differ significantly from those on Earth because of the distinct heat transfer efficiency and pattern from the heated surface to the surrounding liquid^22–29^. Recently, some primary experimental observations of surface bubble generation by pool boiling in the space microgravity environment were reported by Ronshin et al. ^30^ They measured the geometries of the surface bubbles and observed the non-linear bubble volume growth, which is different from the linear bubble volume growth observed on Earth^12^. The Marangoni flow around the surface bubble and its influence on the boiling heat transfer in the space microgravity environment were investigated in Refs. ^31,32^ The authors found that the Marangoni effect was more significant and the flow pattern was different in the space microgravity environment, which changed the temperature profile around the bubble and resulted in a higher bubble growth rate. In addition to bubble growth, the collapse, detachment, coalescence, and dispersion of bubbles in liquid under microgravity were also studied in previous works^33,34^.

The nucleation and growth of surface bubbles involve a complex interplay of physical phenomena. A comprehensive understanding of these phenomena requires consideration of multiple disciplines, including mass transfer, gas diffusion, fluid mechanics, thermodynamics, etc^35–37^. Therefore, precisely predicting the overall dynamics of a surface bubble using numerical methods can still be very challenging given the limitations in the model geometries, mesh density, and time step size, as well as the approximations in the physical properties of fluid that we usually employed in these simulations^23,38–40^. On the other hand, despite the insights gained from studying bubble dynamics in the space microgravity environment can benefit many important practical applications, experimental studying surface bubble nucleation and growth dynamics is still uncommon due to the technical challenges in conducting experiments in the unique environmental conditions associated with the high experimental costs^41–43^. One of the major fluid flows in pool boiling heat transfer that changes dramatically from terrestrial gravity to a microgravity environment is thermal convective flow^44–48^. Thermal convective flow is produced by the temperature-gradient-induced density gradient in a fluid. The hotter fluid with lower density rises upward while the colder fluid moves downward, driven by the buoyancy force on Earth. However, the buoyancy force in the space microgravity environment is almost negligible due to the microgravity environment, largely reducing the significance of the thermal convection effect. Owing to the difficulties in experimental approaches, the detailed analysis of how the reduced thermal convection flow influences surface bubble nucleation and growth dynamics in the space microgravity environment and their comparisons to the terrestrial experiments are still lacking. In this work, we carried out experiments onboard the International Space Station (ISS) to study the nucleation and growth of surface bubbles on heated substrates with different microstructures under microgravity. Videography revealed that surface bubbles nucleated and grew much faster in space than those on Earth. Our thermofluidic simulations attributed the interesting bubble dynamics in the space microgravity environment to the effects of the reduced thermal convective flow.

We also studied the influence of the characteristic length of surface microstructures on bubble nucleation. Bubble dynamics on nano/micro-structures pre-decorated surfaces have already attracted much research attention in recent decades^49–52^. For instance, Liu et al. ^53^ and Chen et al. ^54^ found the densities and geometries of the gold nanopillars and micro-pyramids on surfaces can significantly influence the collective input heating power, and thus affect the nucleation time of surface bubbles. Dong et al. ^55^ has revealed that the characteristic length and surface wettability of microstructures can exert notable influence on the nucleation of surface bubbles. Specifically, when the characteristic length of a microstructure approaches a range of 5 to 100 times smaller than the bubble radius, the microstructure’s impact becomes markedly pronounced, leading to enhanced surface bubble nucleation. Conversely, when the characteristic length surpasses this range, the dominant factor shifts to surface wettability. In our experiments, we utilized substrates with varying porosities yet similar wettability, each characterized by microstructure characteristic lengths ranging from approximately 100 to 500 µm. The surface bubbles in this work typically have a radius of a few millimeters, exceeding the microstructure characteristic lengths by about one order of magnitude. Therefore, our study focuses on the influence of microstructure characteristic length on bubble nucleation process rather than surface wettability. We found the microstructures function as fins to enhance the cooling of the surface. With finer microstructures enabling better surface-to-liquid heat transfer, which cools the surface temperature, the bubble nucleation takes longer. These results revealed interesting physics and may push the boundaries of the knowledge in this field to benefit many space and terrestrial applications, such as phase change cooling and sensing^56–58^.

## Results and discussion

### Overview of terrestrial and space experiments

**Fig. 1:**
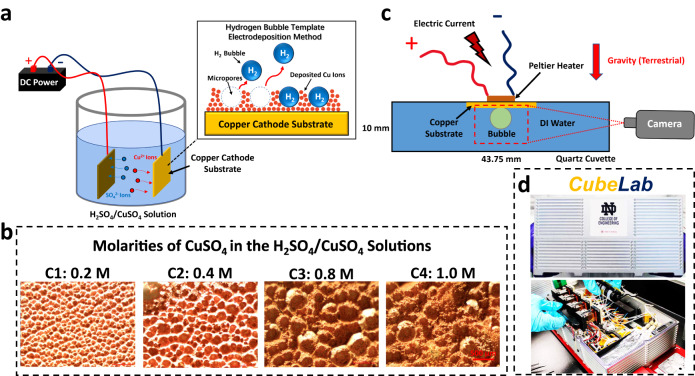
Experimental setup and samples used in the experiments. **a** The schematic of the hydrogen bubble template electrodeposition method used to fabricate the microstructured Cu substrates. **b** The optical images showing the Cu substrates (C1 ~ C4) with different porosities, using the molarities of CuSO_4_ from 0.2 to 1.0 M, respectively. **c** The schematic of the setup to generate surface bubbles by surface heating and monitor their nucleation and growth processes. **d** The integrated instrument, ‘CubeLab’, developed by Space Tango for this project.

A set of Cu microstructured substrates was employed to transfer heat into the boiling system. These substrates were created using the hydrogen bubble template electrodeposition technique (Fig. 1a and the Methods section)^59–62^. Through the application of a DC power supply to both the Cu cathode and anode substrates submerged in an H_2_SO_4_/CuSO_4_ solution, Cu^2+^ ions migrated due to the external electric field. These ions were subsequently deposited onto the Cu cathode substrate, which would later generate surface bubbles in pool boiling experiments. By varying the molarity of CuSO_4_, we controlled the porosity of the microstructures on the Cu substrates, resulting in distinct structure characteristic lengths. This project investigated four microstructured Cu substrates, denoted as C1 (0.2 M), C2 (0.4 M), C3 (0.8 M), and C4 (1.0 M) based on the molarities of CuSO_4_. As depicted in the optical images in Fig. 1b, the microstructure’s characteristic length increased with higher CuSO_4_ molarities. The experimental setup is shown in Fig. 1c with more details included in the Methods section. We note that the bubble was generated on the Cu substrate attached to the top inner wall of the cuvette while gravity is downward in the terrestrial experiments (see Fig. 1c). Given the constraints imposed by electric power limitations in our compact instrument aboard the ISS, we faced restricted heating power for the boiling system. Under the limited heating powder, when the Cu surface is facing upward, the natural convection will quickly cool down the surface and thus significantly delay the bubble nucleation time way beyond the video recording capacity for our ISS experimental system. Therefore, this surface orientation was deliberately chosen to face downward to expedite the bubble generation process in the terrestrial experiments. Additionally, it also prevents bubble detachment from the surface due to buoyancy. Then, the whole setup was integrated into a ‘CubeLab’ instrument box, developed by Space Tango (Fig. 1d). Importantly, all the experiments in the space microgravity environment were conducted inside the NASA ISS, which means the experimental condition was ambient pressure rather than vacuum.

### Comparison of the terrestrial and space bubble dynamics

**Fig. 2:**
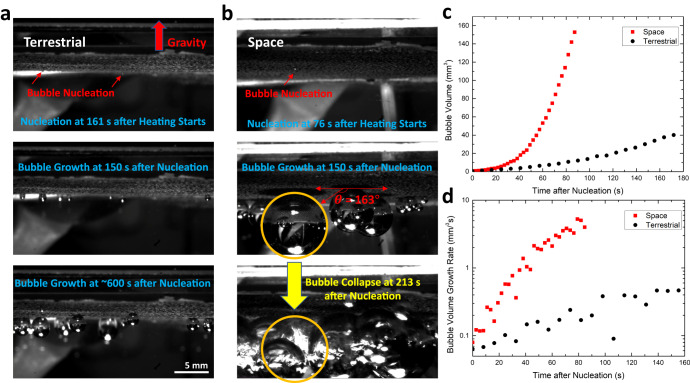
Surface bubble nucleation and growth dynamics. The snapshots showing the moments of surface bubble nucleation (upper), growth (middle) and the final phase (lower) at the end of the video in terrestrial **a** or space microgravity **b** condition. The measured surface bubble contact angle is provided in the middle panel of **b**. **c** The volumes of space (red) and terrestrial (black) bubbles as a function of time after nucleation. **d** The volume growth rates of space (red) and terrestrial (black) bubbles as a function of time after nucleation in log scale.

Figure 2a and b show several typical frames from the recorded videos in the terrestrial and space microgravity environment (also see Supplementary Movies 1 and 2). The upper, middle and lower panels are the moments of a surface bubble nucleation, growth and the final phase at the end of the video, respectively. In Fig. 2a and b, the heating power, cuvette setup and volume, and air concentration of DI water were kept the same in the experiments on Earth and in space. The C4 substrate with the largest characteristic length (see Fig. 1b) was used in both cases. Therefore, the only difference between the experiments in the terrestrial and space microgravity environment is whether gravity influenced the fluid flow during the bubble formation. Comparing the snapshots of the experiments in the terrestrial (Fig. 2a) and space microgravity (Fig. 2b) conditions, we first found that the nucleation of space bubble was faster than the terrestrial bubble (upper two panels). The bubble nucleation occurred at around 76 s in the space microgravity environment after we started heating. In comparison, nucleation took about twice the heating time and started at ~161 s in the terrestrial condition with the same experimental setup. Besides, as we can see in the middle two panels, the space bubbles were much larger than the terrestrial bubbles at the same time (150 s) after nucleation, which means the growth of space bubbles was also much faster. Finally, it is interesting that the space bubbles suddenly collapsed after the heating process lasted for a certain period (~213 s), but the terrestrial bubbles never reached that phase throughout the whole heating process that lasted for ~600 s (lower two panels). It is important to highlight that two sets of experiments were conducted under identical experimental setups and settings on the ISS through two separate missions by SpaceX Cargo Dragon 22 and Northrop Grumman Cargo Mission 17. Both sets of experiments yielded very similar surface bubble nucleation and growth dynamics for the same type of substrates fabricated using the CuSO_4_ molarity (i.e., samples have similar characteristic lengths). Supplementary Movie 3 demonstrates that the nucleation time (~70 s) for another C4 substrate sample during the second flight to the ISS remains similar to the results observed in the first flight as depicted in Fig. 2b and Supplementary Movie 2. This consistency suggests that the irregularities or the exact surface morphology on the microstructured substrates should not be the primary driver behind the observed surface bubble dynamics.

To further quantify the difference in surface bubble growth, we plotted the volumes of space and terrestrial bubbles as a function of time after nucleation. The bubble radius was measured from the image against a pre-defined scale bar, and the measurement uncertainty is ±0.02 mm (see the Supplementary Information, Supplementary Note 4, for the details). As shown in Fig. 2c, the volume of the terrestrial bubble (black) grows slowly with time, consistent with previous findings on dissolved gas-repelling-induced bubble growth^12,13^. However, the volume of the space bubble (red) grows much faster, and the size can reach about 10 ~ 20 times larger than the terrestrial bubble. Interestingly, the volume growth of the space bubble is nonlinear with time, suggesting a different surface bubble growth mechanism in the space microgravity environment. We will further investigate it in the following section (Fig. 5). In Fig. 2d, we plotted the volume growth rates of space and terrestrial bubbles with heating time in log scale. The volume growth rate of the terrestrial bubble is relatively stable during most of the growth stage, but the growth rate of the space bubble has increased by ~2 orders of magnitude during the same period. It finally reaches ~30 times greater than the volume growth rate of the terrestrial bubble before collapsing.

### Thermofluidic surface bubble nucleation simulation

**Fig. 3:**
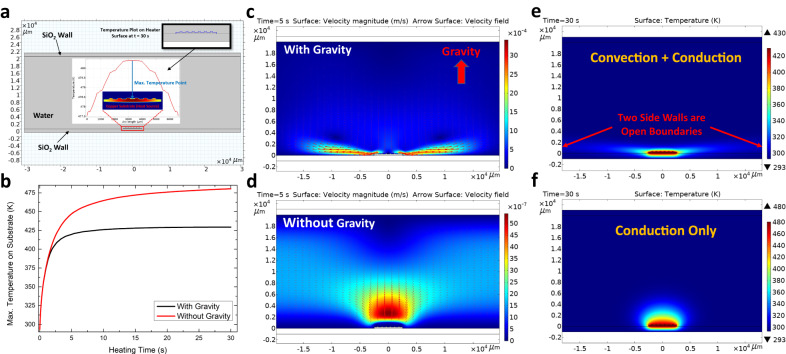
Thermofluidic surface bubble nucleation simulations. **a** The model used in the thermofluidic surface bubble nucleation simulations. The insert shows the temperature plot along the heating surface indicating the maximum surface temperature locates at the center. **b** The calculated maximum substrate surface temperatures as a function of heating time in the terrestrial (black) and space microgravity (red) conditions. The simulated fluid velocity fields at t = 5 s in the terrestrial **c** and space microgravity **d** conditions. The simulated temperature profiles at t = 30 s in the terrestrial **e** and space microgravity **f** conditions.

To understand the experimental findings and compare the different surface bubble dynamics from Earth to space, we performed thermofluidic simulations using the finite element method to help analyze the bubble nucleation and growth processes. The model used to simulate the nucleation of space and terrestrial bubbles is shown in Fig. 3a, with more details of the model setup and simulations included in the Supplementary Information, Supplementary Note 1. The flow effect, heat conduction and thermal convection were included in our transient model, and all the geometries were built according to the actual dimensions of the experimental setup. In this 2D model, a large box of water (60 mm $\times$ 20 mm) is sandwiched by two thin layers of solid SiO_2_ (60 mm $\times$ 1 mm). A thin layer of microstructured Cu substrate (5 mm $\times$ 0.2 mm) is immersed in the water, which is the heating source of the boiling system. In striving for a balance between computational feasibility and accuracy, we have chosen to simplify the representation of the heating substrate to facilitate a comparison of the temperature profiles and flow fields between the cases with and without gravity. The geometry of the microstructure on the Cu substrate was built according to the characteristic length of the C4 substrate (Fig. 1b). We can mimic the terrestrial and space microgravity conditions by switching on or off gravity effect in these simulations, respectively.

A simulated temperature profile of the Cu substrate is shown in the insert of Fig. 3a. Temperature is distributed symmetrically along the horizontal axis, while the maximum surface temperature is located at the center of the substrate. Surface bubble nucleation typically commences when the surface temperature of the heating substrate attains the nucleation temperature. While we did not determine the exact nucleation temperatures, they should fall within a range between the boiling temperature (~100 °C) and the spinodal temperature (~300 °C), as reported in previous literature^63,64^. Consequently, we have constructed a graphical representation illustrating the maximum substrate surface temperature as a function of heating time, employing this approximate scale as a reference, as presented in Fig. 3b. The maximum substrate surface temperature increases much faster in the space microgravity environment (red), and it can reach ~50 K higher than the terrestrial case after the heating process lasts for ~15 s. It indicates that the terrestrial model needs much longer heating time to reach the nucleation temperature, i.e., nucleation time, compared to the space microgravity model. Although the amplitudes of the nucleation times in the simulations are different from the actual experiments due to limitations in model geometries (e.g., the exact surface morphology, 2D simulation), these simulation results nevertheless reproduce the experimental trend that surface bubble nucleates much faster in the space microgravity environment as shown in Fig. 2a and b (upper two panels).

The absence of gravity is the key reason why substrate surface temperature increases faster in space than that on Earth (Fig. 2c and d). As we discussed above, the density gradient induced by the temperature gradient can lead to thermal convection in the gravity field. This is evidenced by the simulated fluid velocity field in the terrestrial model shown in Fig. 3c. Strong circulation is formed on each side of the heated substrate with opposite directions, and the magnitude of flow velocity can be as high as ~10^-3 ^m/s. The feature of fluid circulation indicates that there is significant thermal convection flow in the liquid^65^. However, due to the absence of gravity, thermal convection does not contribute to the fluid flow field in the space microgravity model (Fig. 3d), leading to the flow velocity dropping by ~3 orders of magnitude to ~10^-6 ^m/s. The weak fluid flow in the space microgravity environment is only due to the expansion of the hotter liquid near the heating substrate^66^. The flow field can influence the temperature profile in the boiling system. In the terrestrial model, the thermal convective circulation will grow increasingly more prominent during the heating process, transferring heat away from the hot substrate to the bulk liquid (Fig. 3e). This makes the substrate surface temperature in the terrestrial model increase slower than the in-space-microgravity counterpart, where heat transfer is dominated by conduction. Thus, heat is more localized around the substrate surface, leading to faster surface temperature rise and hence earlier bubble nucleation (Fig. 3f).

### Surface bubble nucleation on different substrates

**Fig. 4:**
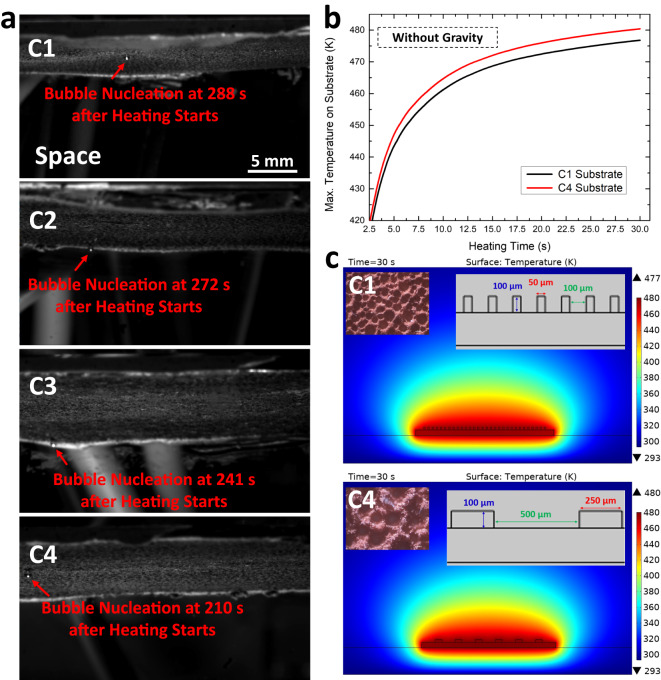
Surface bubble nucleation on different substrates. **a** The snapshots showing the surface bubble nucleation on the substrates with different characteristic lengths. **b** The simulated maximum substrate surface temperatures as a function of heating time on C1 (black) and C4 (red) substrates. **c** The simulated temperature profiles at t = 30 s on C1 (upper) and C4 (lower) substrates in the space microgravity environment. Inserts show the characterization image and dimensions of the surface microstructures.

We also studied the influence of surface microstructure on bubble nucleation time. As shown in Fig. 1b, we prepared four microstructured substrates with a range of characteristic lengths (100 ~ 500 nm). We conducted a boiling experiment using each of these substrates in the space microgravity environment while all the other experimental parameters and setup were kept the same. The heating power was tuned down in this set of experiments compared to Fig. 2 to magnify the difference in the nucleation times among these substrates. As shown in Fig. 4a, the nucleation time decreases monotonically as the characteristic length increases (also see Supplementary Movies 4 and 5). That is to say, the finer structure usually requires a longer time to nucleate. To understand this, we repeated the thermofluidic simulations to compare the surface temperature profiles of the finest (C1) and the coarsest substrate (C4).

The microstructures were modeled as fins standing on the substrates with the spacing set as the average characteristic length of the micropores obtained from the experimental characterization of the corresponding substrates (Fig. 1b). In the simulations, the heat generation rate of the two substrates was kept the same, and no gravity was considered. Figure 4b shows that the maximum substrate surface temperature increases as a function of heating time on each of the two substrates. Although the temperature difference between the two substrates is not as significant as that between the terrestrial and space microgravity models in Fig. 2, we can still find that the C1 substrate needs slightly longer time to reach the nucleation temperature than the C4 substrate. The simulated temperature profiles around the heating substrates are shown in Fig. 4c. Since both substrates were simulated in the space microgravity setting, the heat from the substrate can only be dissipated by conduction. Those microstructures on substrate surfaces can behave as fins to enhance heat conduction – an effect seen extensively for convective interfaces but also observed in conductive interfaces^67^. Compared to the coarser surface, the finer surface has a denser fin structure, resulting in better heat conduction across the interface^68,69^, which helps cool the surface more efficiently than the coarser surface. We note that bubble nucleation can also depend on the nucleation site and the trapped gas in the microstructures^70–72^. However, the finer structures are expected to provide more nucleation sites and more easily trap gas than the coarser structures^20,73^, which thus should not be the root cause for the observed trend in nucleation times.

### Comparison of surface bubble growth in space and on ground

In this section, we will discuss the different surface bubble growth behaviors between the terrestrial and space microgravity experiments presented in Fig. 2c and d. There are two major stages in surface bubble growth^12^. Stage I is an explosive growth due to the vaporization of the liquid surrounding the nucleation site on the substrate, and stage II is a slower growth phase due to the expelling of dissolved gas from the liquid surrounding the bubble. Usually, stage I takes a much shorter time (~10 ms) than stage II, with the latter generally lasting for seconds to minutes. In the stage I growth, the volume of the surface bubble *V* is proportional to the square root of time, *t*^0.5^, by the equation^12^:

1 $$V(t)\propto {\left(\frac{P}{\rho \Lambda }\right)}^{1/2}{\cdot t}^{1/2}$$

where *P* is the heating power of the boiling system, $\rho$ and $\varLambda$ are the density and latent heat of water, respectively. In the stage II growth, the volume of the surface bubble is proportional to time, *t*, by the equation:^12^

2 $$V\left(t\right)=\frac{1}{3}\cdot \left(\frac{{R}_{{\rm{g}}}T}{{M}_{{\rm{g}}}{P}_{{{\infty }}}}\frac{{C}_{{{\infty }}}}{{C}_{{\rm{s}}}}|\frac{d{C}_{{\rm{s}}}}{{dT}}|\frac{{fP}}{{c}_{{\rm{w}}}\rho }\right)\cdot t$$

where ${R}_{{\rm{g}}}$ is the gas constant, *T* is the local temperature of the water surrounding the bubble interface, ${M}_{{\rm{g}}}$ is the molecular mass of air, ${P}_{{{\infty }}}$ is ambient pressure, *C*_s_ is the local air solubility of the water surrounding the bubble interface, *C*_∞_ is the gas saturation far away from the bubble, $f$ is the heating efficiency of the boiling system, and ${c}_{{\rm{w}}}$ is the specific heat capacity of water.

**Fig. 5:**
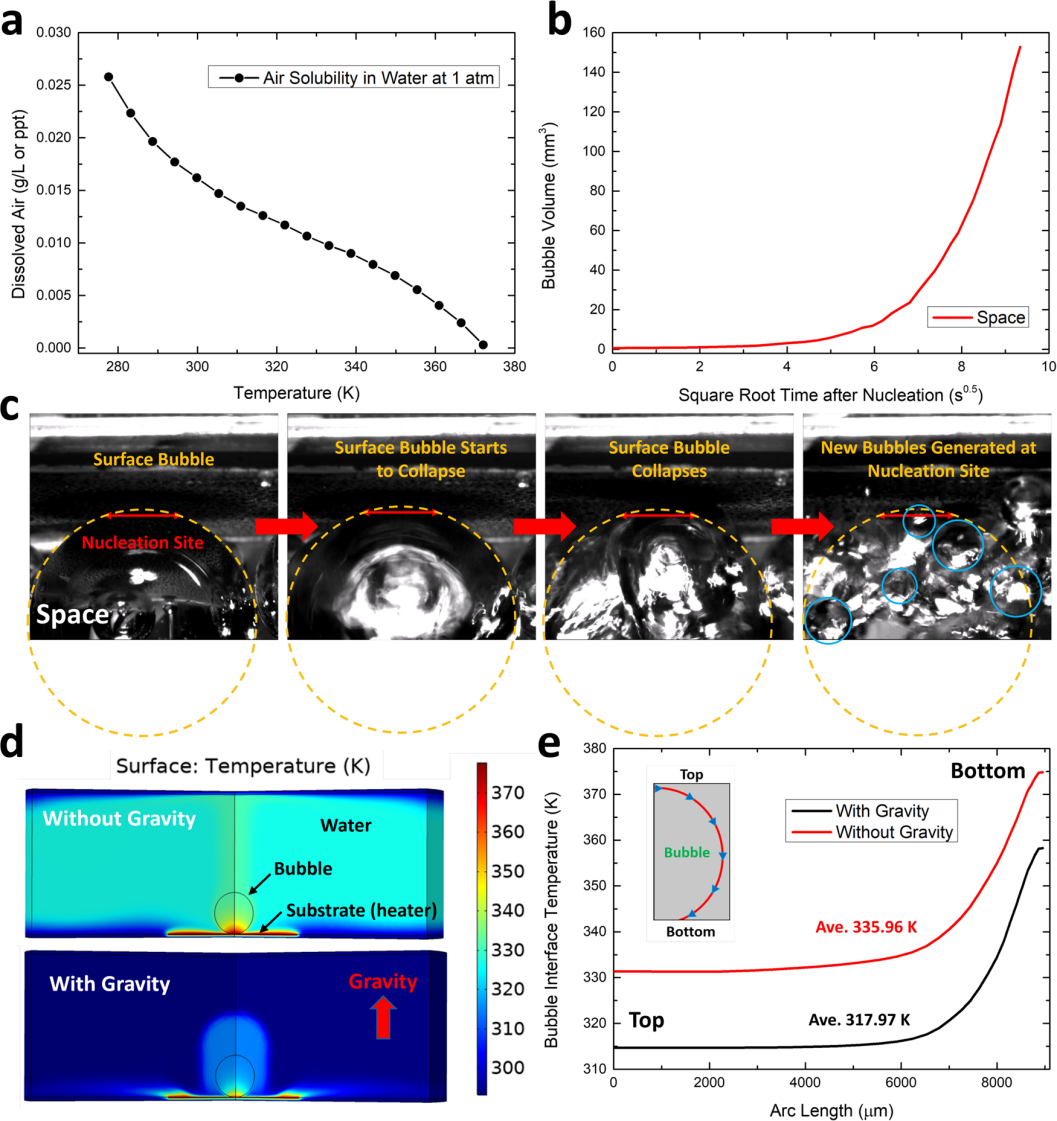
Thermofluidic surface bubble growth simulations. **a** Air solubility in water at 1 atm pressure as a function of temperature. **b** The space bubble volume as a function of the square root of heating time after nucleation, *t*^0.5^. **c** The snapshots showing the collapse of a surface bubble (yellow circle) in the space microgravity environment. After collapse, many smaller bubbles (blue circles) were generated at the nucleation site (red line) on the substrate. **d** The simulated temperature profiles around space (upper) and terrestrial (lower) bubbles. **e** The simulated bubble interface temperature as a function of arc length in the space microgravity (red) and terrestrial (black) models. The insert shows the plotting path along the bubble interface.

Considering that the time resolution of our camera is only ~9 ms, the period of bubble growth that can be resolved in our videos should be mainly the stage II growth. This is also evidenced by the bubble volume growth plots in Fig. 2c that there is no steep explosive growth period (stage I) at the very beginning of the bubble life as reported in previous works^12,13,18^. The volume growth rate of the stage II bubble is described in Eq. (2), in which there are three variable terms: local temperature *T*, local air solubility ${C}_{{\rm{s}}}$ and $\left|\frac{d{C}_{{\rm{s}}}}{{dT}}\right|$. The air solubility in water decreases as temperature increases as shown in Fig. 5a^74,75^. As we can see, the relation between *T* and ${C}_{{\rm{s}}}$ is nearly linear in the experimental temperature range from room temperature (~293 K) to the boiling point (~373 K), which means $|\frac{d{C}_{{\rm{s}}}}{{dT}}|$ does not change significantly in this range, leaving the only two major variables to be *T* and ${C}_{{\rm{s}}}$. For the terrestrial bubble, the volume growth is much slower than the space bubble (Fig. 2c (black) and refs. ^12,13,18^), suggesting that the vibration of volume growth rate is much smaller. This means the local temperature in the water boundary layer^76^ around the surface bubble interface should be almost constant during the stage II growth on Earth^12^. However, as we can see in Fig. 2c and d (red), the bubble volume growth is nonlinear in the space microgravity environment, i.e., the growth rate increases with heating time. Such a dramatically different behavior can be from two possibilities: the local temperature around the bubble interface keeps increasing during the stage II growth in the space microgravity environment, or space bubble growth is dominated by water vaporization (stage I growth) instead of expelling dissolved air (stage II growth). Bubble volume growth dominated by the water vaporization around the bubble interface follows Eq. (1), which indicates the volume should be linearly proportional to *t*^0.5^. We plotted the space bubble volume as a function of the square root of heating time, *t*^0.5^, in Fig. 5b. It is obvious that no linear relation can be found between *V* and *t*^0.5^, meaning that the space bubble does not follow the stage I growth pattern. These analyses suggest that the space bubble is also a stage II air bubble but with increasing local temperature during the growth process.

We know that space bubbles also mainly consist of air, but how high the local temperature around the bubble interface should be in order to support such faster bubble growth (~30 times at the end of the growth stage) and larger volume compared to terrestrial bubbles? While Eq. (2) details the volume growth of stage II air bubble, it is important to acknowledge that we cannot experimentally measure or precisely calculate the exact value of the local air solubility or heating efficiency *f* of the external heater used in the pool boiling setup. Consequently, direct calculation of local temperature from the measured bubble volume growth rate is unattainable. Nevertheless, it is crucial to emphasize that the heating efficiency of the heater should remain consistent between the space microgravity and terrestrial experiments as they used the same experimental setup and heater settings. As a result, the ~30 times higher bubble volume growth rate in the space microgravity environment (Fig. 2d) is likely only contributed by the higher local temperature and its induced lower local air solubility. The air solubility-temperature relation plot in Fig. 5a shows that the temperature can only increase by ~1.3 times from room temperature (~293 K) to the boiling point (~373 K). That is to say, the local temperature of the space bubble keeps increasing during the bubble growth, which eventually leads the temperature around the bubble interface near the heater surface to approach the boiling point at the end of the growth stage (before collapse).

The elevated local temperature in the microgravity condition, as a result of the absence of thermal convective flow, can indeed have significant effects on bubble behavior. This high local temperature can lead to a drastic decrease in the local air solubility by over 20 times and approach near-zero values. This change in solubility is the key factor contributing to the extremely high bubble volume growth rate observed at the end of the growth stage compared to terrestrial bubbles, as demonstrated in Eq. (2). However, it is essential to note that the high local temperature in the microgravity environment may also have adverse effects on bubble stability, making bubbles more prone to collapse^77^. This phenomenon is supported by the visual evidence provided in Fig. 5c and Supplementary Movie 6, which show the moments of surface bubble collapse in microgravity. In these snapshots, many smaller bubbles are generated at the original nucleation site of the collapsed surface bubble. These smaller bubbles are rapidly ejected from the nucleation site and eventually disperse throughout the surrounding liquid, a behavior that suggests nucleate boiling occurring at the nucleation site^78,79^. The ejection of these smaller bubbles from the nucleation site may be attributed to the vapor nature of the bubbles generated by nucleate boiling.

We conducted thermofluidic simulations to compare the bubble interface temperatures on Earth and in space using the model shown in Fig. 5d (the Supplementary Information, Supplementary Note 2). In the model, an air surface bubble with a radius of 3 mm (the size was obtained from Fig. 2c) was added to the nucleation model used in Fig. 3. The contact angle of the bubble on top of the heating substrate was built according to ref. ^12^ Similar to the nucleation simulations, the only difference between the terrestrial and space microgravity models is whether gravity was considered. Based on the analysis above, we set the bubble interface temperature at the bottom of the bubble around the nucleation region to reach the boiling point (373 K) in the space microgravity environment. Then we conducted the simulation of the terrestrial model with the same heating power and efficiency as the space microgravity model. The simulated steady-state temperature profiles of space and terrestrial bubbles are shown in Fig. 5d. We also plotted the bubble interface temperatures from the top to the bottom of the bubble along its interface in Fig. 5e. The highest temperature is located at the bottom of the bubble in both cases, which contributes to the major portion of the bubble volume growth rate due to the significantly higher temperature and its induced lower air solubility. The interface temperature of the space bubble (red) is ~20 K higher than the terrestrial bubble (black). This is again because the heat transfer is much faster in the terrestrial model where convection and Marangoni flow^80–82^ can quickly transfer the heat away from the substrate surface (see Fig. 5d). As a result, the key reason leading to the fast bubble growth and large bubble volume in the space microgravity environment is the high local temperature and its induced low air solubility near the bubble base. Since the local temperature can be as high as the water boiling point at the end of the growth stage, we believe that the ratio of vapor to air inside the space bubble right before collapse should be significantly higher than that of the terrestrial bubble.

In summary, the nucleation and growth dynamics of surface bubbles on Earth and in space have been systematically investigated and compared both experimentally and theoretically in this work. Due to the weak gravity field in space, the thermal convective flow is negligible compared to the case on Earth, which results in a much higher local temperature around the bubble nucleation site. Such a high local temperature can significantly accelerate the surface bubble nucleation and reduce the heating time required by about half. Moreover, we found the local temperature around the bubble interface can be close to the water boiling point and lead to extremely fast bubble growth (~30 times faster than terrestrial bubble) and large bubble volume in the space microgravity environment. We also demonstrated that the finer the microstructures on the heating substrate, the longer the bubble nucleation time. This is mainly because the microstructure will behave as the fin structures to enhance heat conduction, and the finer fin structure has higher heat conduction efficiency. These results provide fundamental insights into surface bubble dynamics, which may provide guidance on designing bubble-based sensors^57^.

## Methods

### Fabrication of the microstructured Cu substrates

As shown in Fig. 1a, the microstructured Cu substrates were fabricated by the so-called hydrogen bubble template electrodeposition method^60,61^. The Cu substrates were prepared as cylinders with a diameter of ~35 mm and a thickness of 0.5 mm before being cleaned sequentially with dilute sulfuric acid, hot dilute caustic solution, and deionized water^62^. A cleaned Cu substrate was then used as the cathode in the setup shown in Fig. 1a. Another Cu plate was placed ~2 cm apart from the cathode substrate to act as the anode. The electrodeposition process was performed in a stationary solution in which the molarity of H_2_SO_4_ was kept at 0.8 M with the molarity of CuSO_4_ ranging from 0.2 M to 1.0 M for different substrates (C1 to C4). A DC power supply (Maynuo 8852) was used for the deposition process, in which the Cu atoms in anode were dissolved into the solution and formed Cu^2+^ ions. These Cu^2+^ ions were driven by the external electric field to move toward and finally deposit onto the cathode Cu substrate. However, if the input current density is high enough, a hydrogen evolution reaction can occur simultaneously with the Cu^2+^ ions deposition process on the cathode to initiate the hydrogen bubble template electrodeposition (Fig. 1a). These abundant hydrogen bubbles generated on the cathode can be used as the templates to construct microporous structures on the cathode Cu substrate. By controlling the molarity of CuSO_4_, we can control the porosities of the microporous structures on the Cu substrates, i.e., the porosity increases as the molarity of CuSO_4_ increases. The deposition process lasted for 60 s each with a current density of 1 A·cm^-2^. It is important to note that the height of the deposited Cu structures predominantly relies on the electrodeposition time^83^. Given that we applied the same deposition time across all our substrates, we can reasonably expect the microstructural heights to be very similar. After the Cu substrates were rinsed with deionized water and dried, they were sintered in a reducing atmosphere at 710 °C for 30 mins to strengthen the microstructure^59^. Supplementary Fig. 8 in the Supplementary Information, Supplementary Note 3, presents the optical microscope images of the Cu substrates both before and after the sintering process. To measure the characteristic lengths associated with each substrate, our initial approach involved fitting the sizes of all the micropores on the substrate with circles. Subsequently, we computed the average diameter of these circles to determine the characteristic length. Additionally, the surface morphologies of the different substrates are measured using optical profilometry (Olympus LEXT OLS4100 confocal microscope)^84^. The images are shown in Supplementary Fig. 9 in the Supplementary Information. Squared mean height (*Sq*) represents the standard deviation of height distribution. The cavity depth was estimated by the Olympus LEXT software from the line profile. It is clear that the finer structures have larger effective surface areas, which informs our modeling study. It is important to emphasize that for this study, we only selected the finest substrate (C1, 0.2 M) and the roughest substrate (C4, 1.0 M) to serve as our simulation cases. This deliberate choice was made to ensure a substantial contrast in characteristic lengths between the cases, facilitating the assessment of the impact of characteristic length on surface bubble nucleation.

### Surface bubble generation and characterization

A fabricated Cu microstructured substrate was attached with thermal epoxy onto the inner wall of a quartz cuvette with the internal dimensions of 10 mm (H) × 20 mm (W) × 43.75 mm (L) and a wall thickness of 1.25 mm (Fig. 1c). We then affix the Peltier heater with a 10 mm × 10 mm surface area on the exterior wall of the quartz cuvette. This design choice simplifies the experimental setups, eliminating concerns about electrical wiring within the liquid and contributing to a more compact instrument configuration considering the reliability and size restrictions in space flight. To ensure the consistency of our experiments and mitigate any potential variations in heat transfer performance resulting from this heater arrangement, the same Peltier heater was maintained in the exact same position without any alterations after the terrestrial gravity experiments, and it was subsequently transported to NASA to flight to ISS for the microgravity experiments. During each boil, the Peltier heater exhibited a voltage of ~4.1 V, with the current settled at ~1.3 A. The heat from the heater was conducted through the quartz wall, epoxy, and eventually to the Cu substrate for the surface bubble nucleation to occur. We conducted each boiling experiment three times for the same substrate to corroborate the reproducibility of the nucleation time. We note the thickness of the epoxy is much thinner than the thickness of the quartz cuvette wall to minimize the interfacial thermal resistance. The Cu substrate was trimmed to fit the inner width of the cuvette, so they are slightly ~20 mm. The imaging process is also depicted in Fig. 1c. The imaging axis of the camera was aligned with a small angle of ~10 degrees to the substrate plane, and a LED background light was used as the illumination source. All videos were captured at 110 FPS and 2 megapixels resolution. We first used the camera to image a grid with an inter-line distance of 1 mm, which was then used as the pixel-to-real-size converter to quantify the actual sizes of the surface bubbles in the videos (see the Supplementary Information, Supplementary Note 4, for the details). The experimental processes were monitored by the control station on Earth, and the recorded videos were downlinked for detailed analysis. The terrestrial experiments were performed in the CubeLab prior to the space launch to ensure that the only difference between the sets of experiments was from gravity.

### Reporting summary

Further information on research design is available in the Nature Research Reporting Summary linked to this article.

### Supplementary information

Supplementary Information

Supplementary Movie 1

Supplementary Movie 5

Supplementary Movie 6

Supplementary Movie 2

Supplementary Movie 3

Supplementary Movie 4

Reporting Summary

## Data Availability

All the data that support the findings of this study are included in the main text and Supplementary Information, as well as the 6 Supplementary Movies. The datasets generated and analyzed during the current study are also available from the corresponding author upon reasonable request.
