# Supplementary material for: Bubble nucleation and growth on microstructured surfaces under microgravity
Source: NPJ Microgravity. 2024 Jan 30;10:13. doi: 10.1038/s41526-024-00352-0 (PMC10827752; doi:10.1038/s41526-024-00352-0)
Supplement: Supplementary file 1 — Supplementary Information [file 41526_2024_352_MOESM1_ESM.pdf]

Supplementary Information for

Bubble Nucleation and Growth on Microstructured Surfaces under Microgravity

Qiushi Zhang[1], Dongchuan Mo[1], Seunghyun Moon[1], Jiya Janowitz[2], Dan Ringle[2], David Mays[2], Andrew Diddle[2], Jason Rexroat[2], Eungkyu Lee[1,*], and Tengfei Luo[1,3,*]

1. Department of Aerospace and Mechanical Engineering, University of Notre Dame, IN, USA

2. Space Tango, 611 Winchester Rd. Lexington, KY, USA

3. Department of Chemical and Biomolecular Engineering, University of Notre Dame, IN, USA

* Corresponding authors: eleest@khu.ac.kr; tluo@nd.edu

Supplementary Note 1. Finite Element Thermofluidic Surface Bubble Nucleation Transient Simulations.

We employed COMSOL Multiphysics to simulate the transient temperature and flow profiles around the Cu substrate in the boiling systems on Earth and in space. The flow effect, thermal conduction and convection (terrestrial model) in liquid are included in our simulations. The details of the model used in our simulations are shown in **Supplementary Figure 1**.

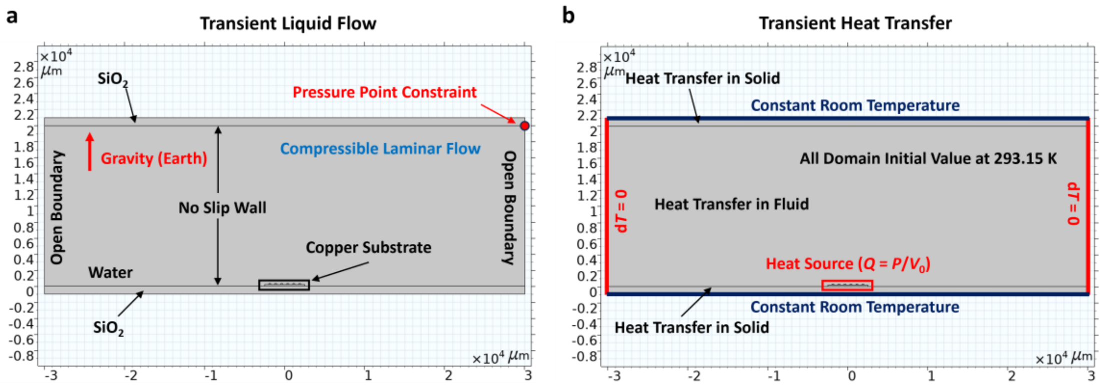

Supplementary Figure 1. Geometry, materials, and boundary conditions used for the finite element thermofluidic transient (a) laminar flow and (b) heat transfer simulations. The material properties are contained in the COMSEL software library.

There are several conditions that have been assumed in our simulations: (1) The liquid flow and heat transfer are both transient. The initial temperature of the whole system is set to 293.15 K. **Supplementary Figures 2** and **3** show the flow velocity fields and temperature profiles of the terrestrial and space microgravity models at $t = 3$, 10 and 20 s, respectively. (2) The liquid water is simulated as compressible and laminar flow (with gravity on Earth, and without gravity in space, see **Fig. 3a** for the direction of gravity on Earth). The Reynolds number can be estimated by multiplying the fluid

velocity by the characteristic length of the flow, and then dividing the result by the kinematic viscosity of water. As illustrated by the flow velocity profile depicted in **Supplementary Figure 2**, the maximum Reynolds number occurs at the location with the highest fluid velocity, which is approximately ~3×10^{-3} m/s. Considering the highest temperature (where the kinematic viscosity of water is approximately ~3×10^{-7} m^2/s at 100 °C), the calculated Reynolds number is approximately 90 for the terrestrial gravity condition.[1] For the space microgravity scenario, the fluid velocity is about three orders of magnitude smaller, resulting in a Reynolds number of less than 0.1. The critical Reynolds number, which signifies the transition from laminar to turbulent flow, typically falls within a broad range spanning approximately 2300 to 4000 for enclosed systems.[2] Therefore, it is reasonable to apply the laminar flow assumption in our simulations.[3–5]

The fluid flow satisfies the following momentum equation, on Earth:

$$\rho \frac{\partial \mathbf{u}}{\partial t} + \rho(\mathbf{u} \cdot \nabla)\mathbf{u} - \nabla \cdot \left(\mu(\nabla \mathbf{u} + \nabla \mathbf{u}^{\mathrm{T}}) - \frac{2}{3}\mu(\nabla \cdot \mathbf{u})\mathbf{I} - p\mathbf{I} \right) - \rho \mathbf{g} = 0 \qquad (1)$$

, and in the space microgravity environment,

$$\rho \frac{\partial \mathbf{u}}{\partial t} + \rho(\mathbf{u} \cdot \nabla)\mathbf{u} - \nabla \cdot \left(\mu(\nabla \mathbf{u} + \nabla \mathbf{u}^{\mathrm{T}}) - \frac{2}{3}\mu(\nabla \cdot \mathbf{u})\mathbf{I} - p\mathbf{I} \right) = 0 \qquad (2)$$

and continuity equation:

$$\frac{\partial \rho}{\partial t} + \nabla \cdot (\rho \mathbf{u}) = 0 \qquad (3)$$

where ρ is the density of water, μ is the dynamic viscosity of water, $\mathbf{u}$ is the velocity vector, p is pressure, t is time, $\mathbf{g}$ is gravity constant, and $\mathbf{I}$ is a 3×3 identity matrix. (3) The SiO_2 cuvette and Cu substrate are considered as rigid solid materials. (4) The heat generation rate Q of Cu substrate is the only heat source of the system, which supplies heat to the liquid water with the following heat transfer equations:

In water,

$$\rho C_{\mathrm{p}} \frac{\partial T}{\partial t} + \rho C_{\mathrm{p}} \mathbf{u} \cdot \nabla T - k_{\mathrm{w}} \nabla^2 T = Q \tag{4}$$

where C_{p} is the heat capacity of water at constant pressure, T is the temperature, k_{w} is the thermal conductivity of water, Q is the heat generation rate by Cu substrate, and in the medium of SiO_2 or Cu,

$$-k_{\mathrm{s}} \nabla T = q \tag{5}$$

where k_{s} is the thermal conductivity of SiO_2 or Cu, and q is the heat flux coming through the liquid/solid interfaces. The boundary conditions used in our simulations are similar to those in ref. [3–5]. The heat generation rate is:

$$Q = \frac{P}{V_0} \tag{6}$$

where P is the heating power and V_0 is the volume of the Cu substrate. To note, the heat generation rate of space microgravity models (C1 and C4 substrates) and terrestrial

model is the same, which is calibrated by letting the max. temperature on the substrate surface in both cases within the nucleation temperature range, between the boiling temperature (~100 °C) and the spinodal temperature (~300 °C), at the end of simulation time (**Fig. 3b**).

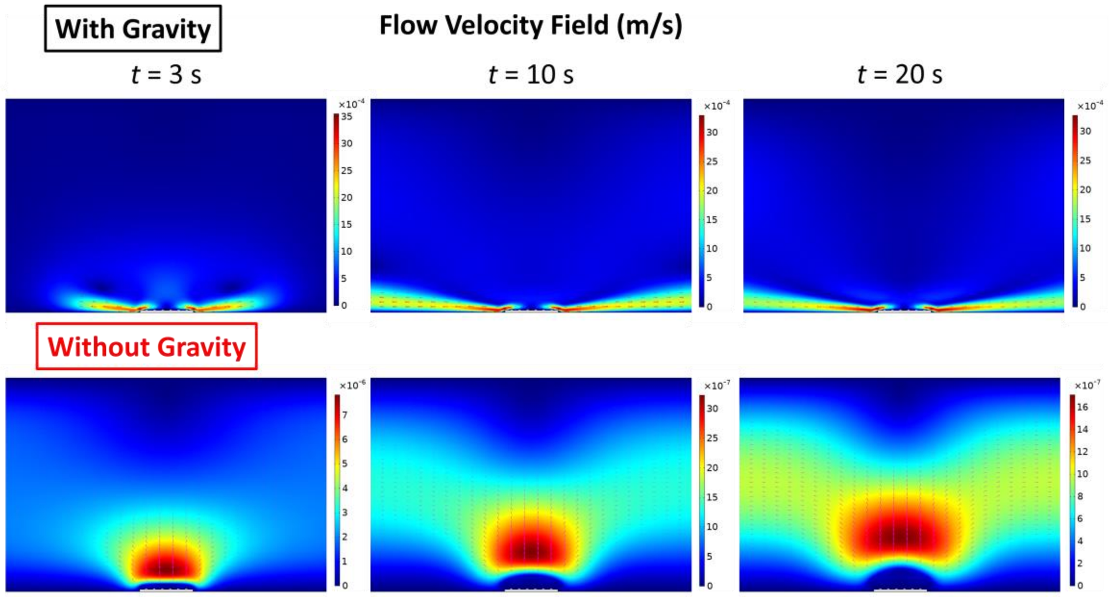

Supplementary Figure 2. The simulated flow velocity fields of terrestrial (upper) and space microgravity (lower) models at t = 3, 10 and 20 s.

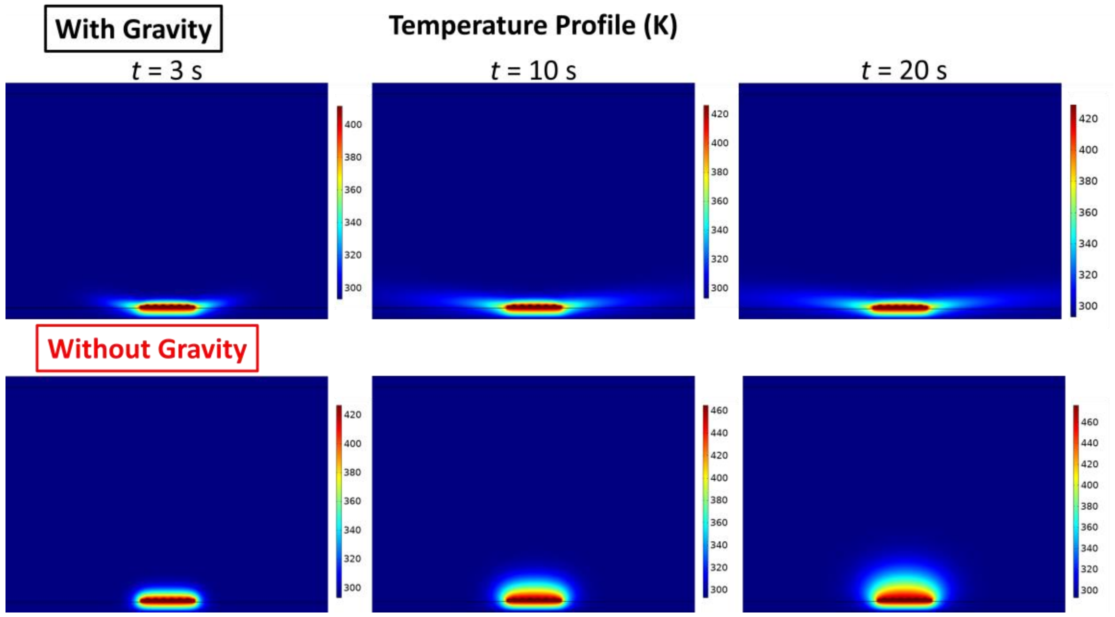

Supplementary Figure 3. The simulated temperature profiles of terrestrial (upper) and space microgravity (lower) models at $t = 3$, 10 and 20 s.

In **Supplementary Figure 4**, Mesh 3 (identified with a star), represents the mesh structure employed in both the simulations with and without gravity. This mesh comprises 79,509 domain elements and 1,993 boundary elements with higher resolution around the heater surface. To ensure the robustness of our simulation results and confirm their independence from the chosen mesh size, we created four additional mesh structures. Two of these meshes (Mesh 1 and Mesh 2) are coarser than Mesh 3, while the other two (Mesh 4 and Mesh 5) are finer, as illustrated in **Supplementary Figure 4**. Subsequently, we conducted simulations of the transient substrate surface temperature as a function of heating time in the condition without gravity. As shown in **Supplementary Figure 5**, the calculated temperature profiles reveal that the temperature variation remains consistently within about 1 K across all five mesh structures that we simulated. This confirms that our simulation results are not contingent upon the specific choice of the mesh size.

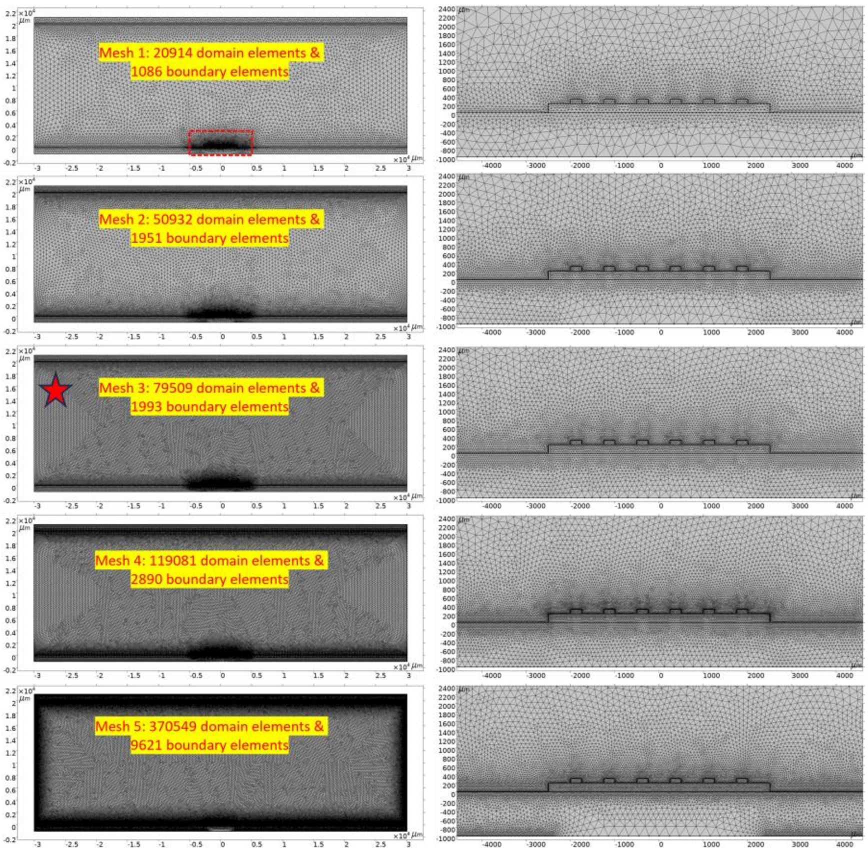

Supplementary Figure 4. The five mesh structures used to verify the independence from the chosen mesh size of our simulation results. Mesh 3 is the mesh structure used in both the simulations with and without gravity (labeled with a red star) in this work. Left panels zoom in the area around the heating substrate (labeled with a red rectangular frame).

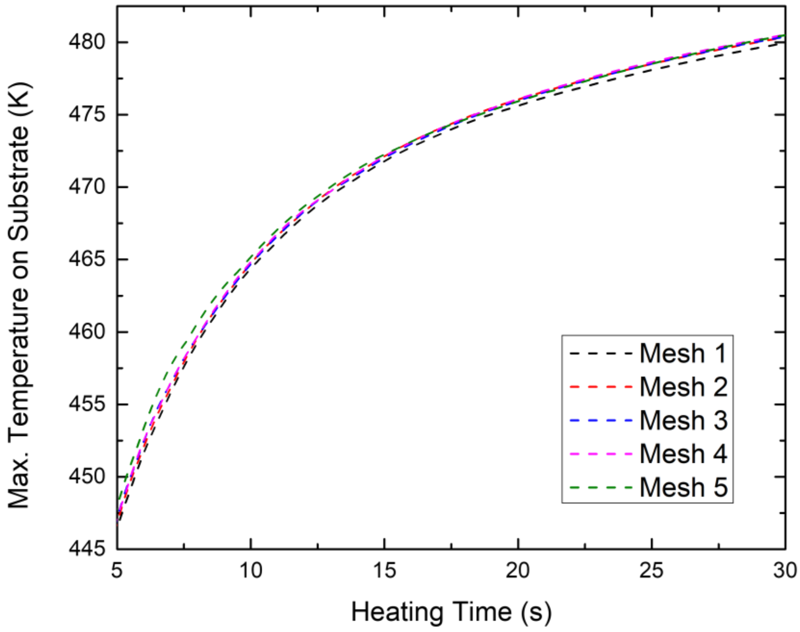

Supplementary Figure 5. The calculated maximum substrate surface temperatures as a function of heating time in the condition without gravity by using the five different mesh structures in **Supplementary Figure 4**.

As the experimental setup described in **Fig. 1c** shows, the heat was provided by a Peltier heater (10 mm × 10 mm) affixed to the outside wall of the cuvette and conducted through the cuvette wall to the Cu substrate (20 mm × 20 mm) for surface bubble nucleation to occur. However, in order to simplify our simulation models, we instead set the Cu substrate as the heat source of the boiling system in this work. To check if the heating effect of our model can represent the real experimental setup, we repeated the simulations of bubble nucleation on Earth and in space while using a more realistic model, i.e., with another layer of Cu (half of the size of the Cu substrate) as the external heater on the outside wall to heat up the Cu substrate in the boiling system (**Supplementary Figure 6a**). As we can see in the simulated temperature profile (**Supplementary Figure 6a**), the heat from the external heater is conducted through the

cuvette wall and mostly localized on the Cu substrate without significant leakage into the cuvette wall that is not covered by the Cu substrate. This is because the heat conductivity of Cu is ~2 orders of magnitude larger than SiO_2. As a result, this indicates that by setting the Cu substrate as the heater of the boiling system, we can still achieve similar heating effect as the experimental system, which is also evidenced by the similar flow field and max. surface temperature plots shown in **Supplementary Figures 6b** and **c**, respectively (compared to **Figs. 3b and c**).

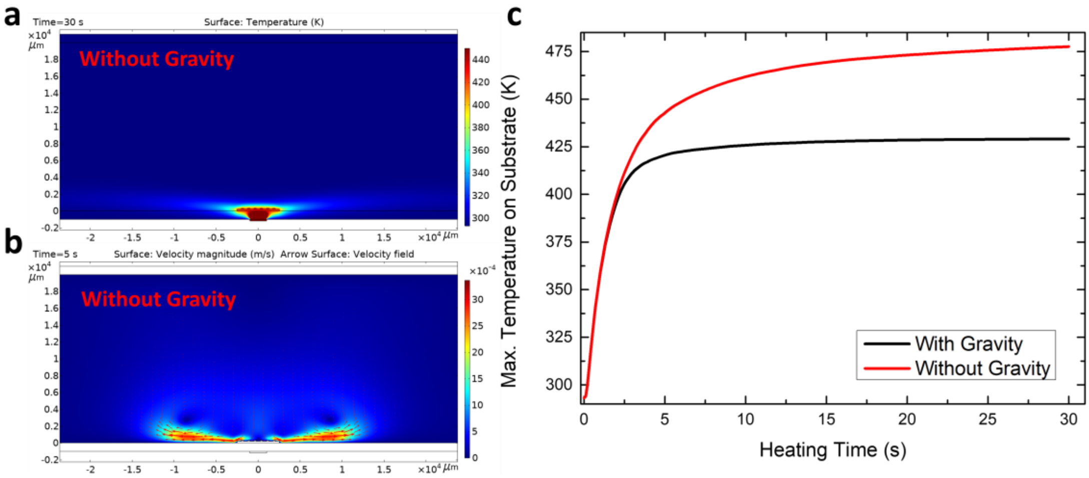

Supplementary Figure 6. The simulated temperature profile at t = 30 s (a) and flow velocity field at t = 5 s (b) of space microgravity model using the realistic model with external heater (compared to **Figs. 3b and c**). (c) The simulated max. surface temperatures on substrate as a function of heating time on Earth (black) and in space (red) using the realistic model.

Supplementary Note 2. Finite Element Thermofluidic Surface Bubble Growth Steady-State Simulations.

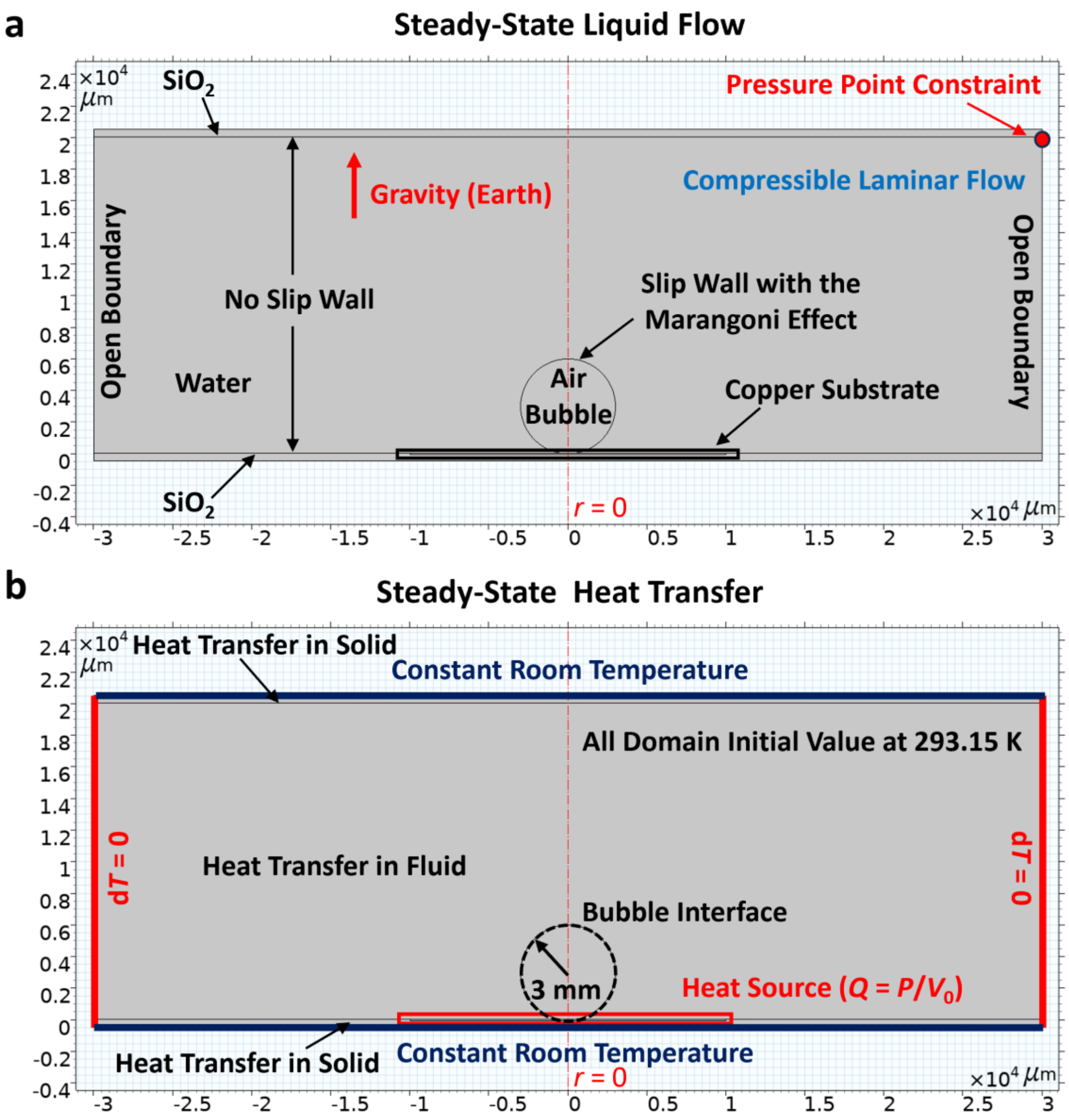

Supplementary Figure 7. Geometry, materials, and boundary conditions used for the finite element steady-state thermofluidic (a) laminar flow and (b) heat transfer simulations with a surface bubble. The material properties are contained in the COMSEL software library.

The model and assumptions of bubble growth simulations are similar to those in the bubble nucleation simulations described in section Supplementary Note 1, but the liquid flow and heat transfer are at steady state here. To note, we opted to model a surface bubble with a fixed size instead of varying sizes and subsequently compared the interface temperature profiles between the terrestrial gravity and space microgravity scenarios, which can significantly reduce computational cost while producing key insight to support our conclusions.[3,4] In liquid water, the momentum equation is:

on Earth,

$$\rho(\mathbf{u} \cdot \nabla)\mathbf{u} - \nabla \cdot (\mu(\nabla \mathbf{u} + \nabla \mathbf{u}^{\mathrm{T}}) - p\mathbf{I}) - \rho \mathbf{g} = 0 \tag{7}$$

, and in the space microgravity environment,

$$\rho(\mathbf{u} \cdot \nabla)\mathbf{u} - \nabla \cdot (\mu(\nabla \mathbf{u} + \nabla \mathbf{u}^{\mathrm{T}}) - p\mathbf{I}) = 0 \tag{8}$$

, and continuity equation is:

$$\rho(\nabla \cdot \mathbf{u}) = 0 \tag{9}$$

where the definitions of variables are the same as those in Supplementary Equations 1, 2 and 3, respectively. The heat transfer equation in water is:

$$\rho C_{\mathrm{p}} \mathbf{u} \cdot \nabla T - k_{\mathrm{w}} \nabla^2 T = Q \tag{10}$$

where the definitions of variables are the same as those in Supplementary Equation 4.

The heat generation rate also follows the same equation as Supplementary Equation 6. An air bubble with a radius of 3 mm was added on top of the Cu substrate (**Supplementary Figure 7**). The gas medium inside the surface bubble, quartz walls and Cu substrate were considered as non-fluidic rigid materials, which have the same heat transfer equation as Supplementary Equation 5. The Marangoni effect exists at the interface of surface bubble (gas/water boundary), which has a slip boundary condition. The Marangoni effect is simulated as:

$$\left[\mu(\nabla\mathbf{u}+\nabla\mathbf{u}^{\mathrm{T}})-\left(p+\frac{2}{3}\mu(\nabla\cdot\mathbf{u})\right)\mathbf{I}\right]\mathbf{n}=\frac{d\gamma}{dT}\nabla_{\mathrm{t}}T \qquad (11)$$

where $\mathbf{n}$ is the normal outward vector to the interface of the bubble, $\frac{d\gamma}{dT}$ is the temperature derivative of the water/gas surface tension, and ∇_{t} is the gradient of the tangent vector to the interface of the bubble. Supplementary Equation 11 describes the thermally induced Marangoni effect relating the normal component of the shear stress along the interface of the bubble to the tangential derivative of the temperature field. In addition, for the compressible fluid considered here in both the terrestrial gravity and space microgravity settings, pressure contribution was also included in the laminar flow.

Supplementary Note 3. The optical images of Cu microstructured substrates.

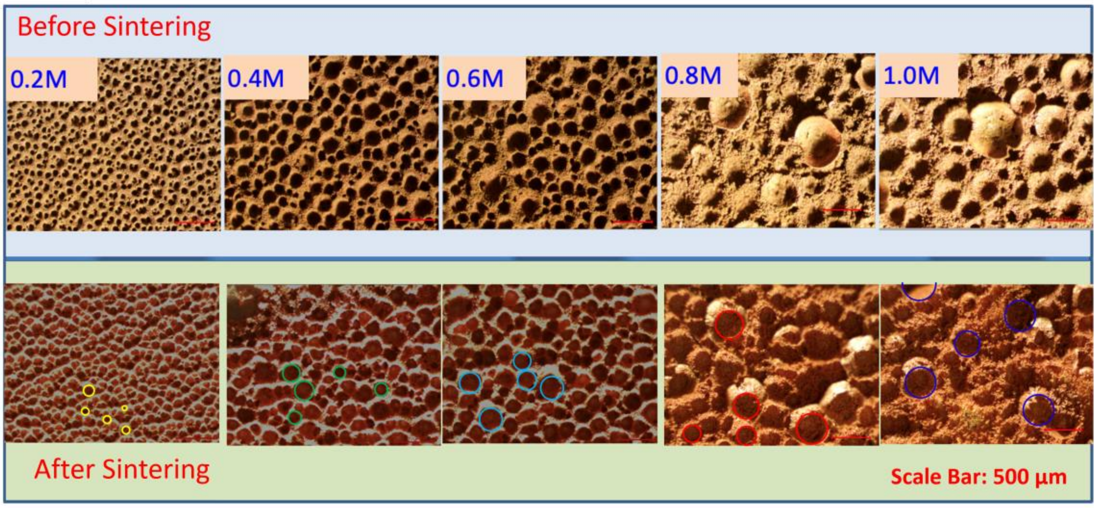

Supplementary Figure 8. The optical images of the Cu microstructured substrates with different molarities of $CuSO_4$ of 0.2 ~ 1.0 M before and after sintering. The colored circles on the images showing the characteristic sizes of the micropores.

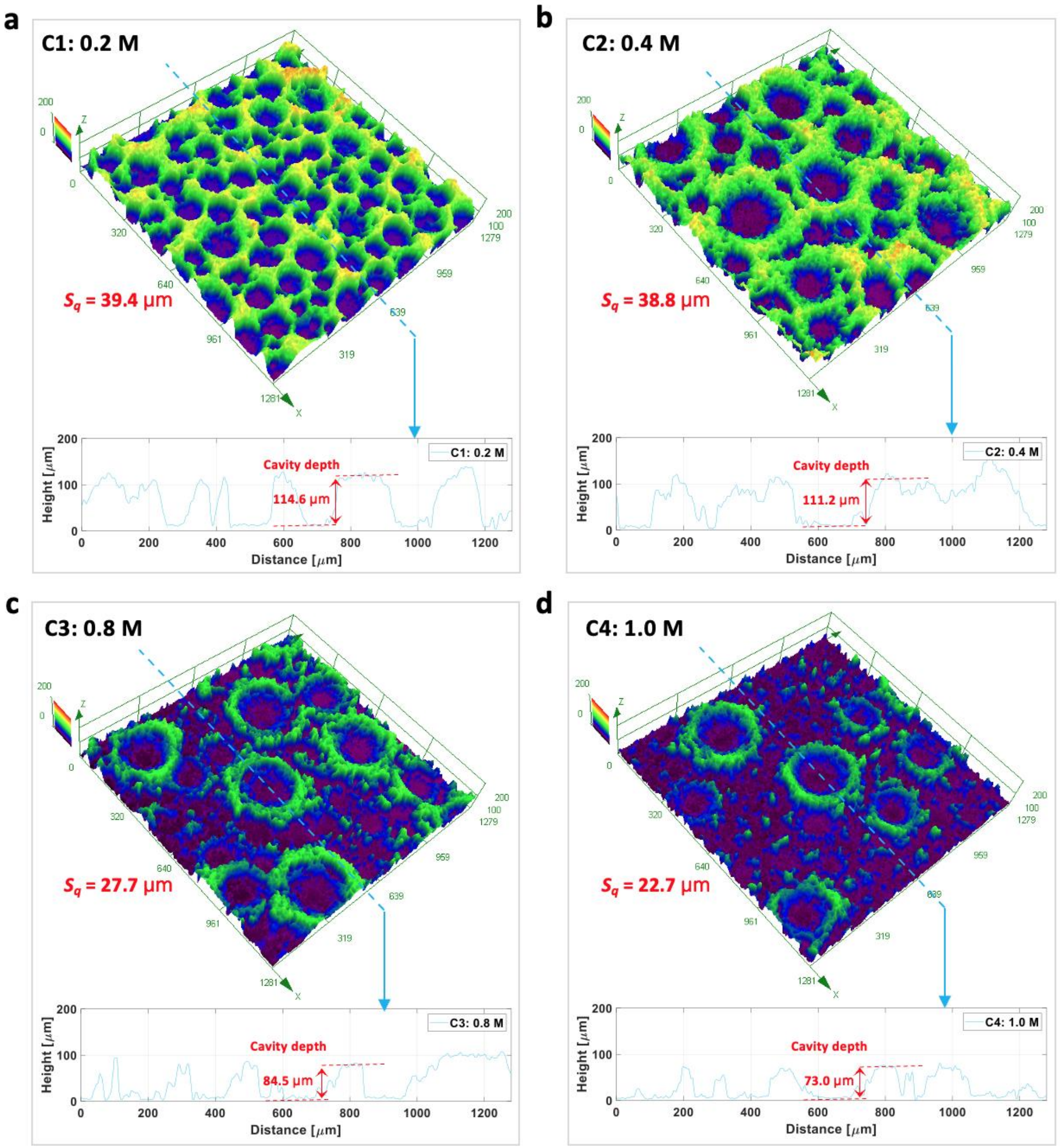

Supplementary Figure 9. Three-dimensional optical profilometry images and line profiles of the 4 different substrates. (a) C1 0.2 M. (b) C2 0.4 M. (c) C3 0.8 M. (d) C4 1.0 M. These images quantitatively show that finer structures have larger effective surface areas.

Supplementary Note 4. The measurement of surface bubble radius.

To measure the radius of the surface bubbles in both terrestrial and microgravity space experiments, we employed the following procedures. First, we utilized the camera to capture images of a grid pattern, as depicted in **Supplementary Figure 10a**, where the known distance between each grid line (red vertical line) is 1 mm. By counting the number of pixels between these grid lines, we established a ratio between the number of pixels in our video and the real size, with 1 mm equivalating to 50 pixels. Subsequently, we employed the same camera settings to capture the surface bubble dynamics on the substrate surface. After recording the video, we used MATLAB to extract individual frames, converting them into JPG files. Within MATLAB, we developed a custom code that included contrast and grayscale adjustments, as well as the utilization of the '*imfindcircles*' function to accurately fit the radius of the bubbles.[3] **Supplementary Figure 10b** illustrates the precision achieved with this circle fitting function. It is important to note that the radius obtained through this MATLAB code is expressed in units of pixels. To convert this to the real bubble radius, we applied the previously determined ratio of pixels to real size. However, it is worth mentioning that the MATLAB code can only provide integer values for pixels, introducing an uncertainty in measuring the bubble radius of ±1 pixel, which equates to an uncertainty of ±0.02 mm in real length unit.

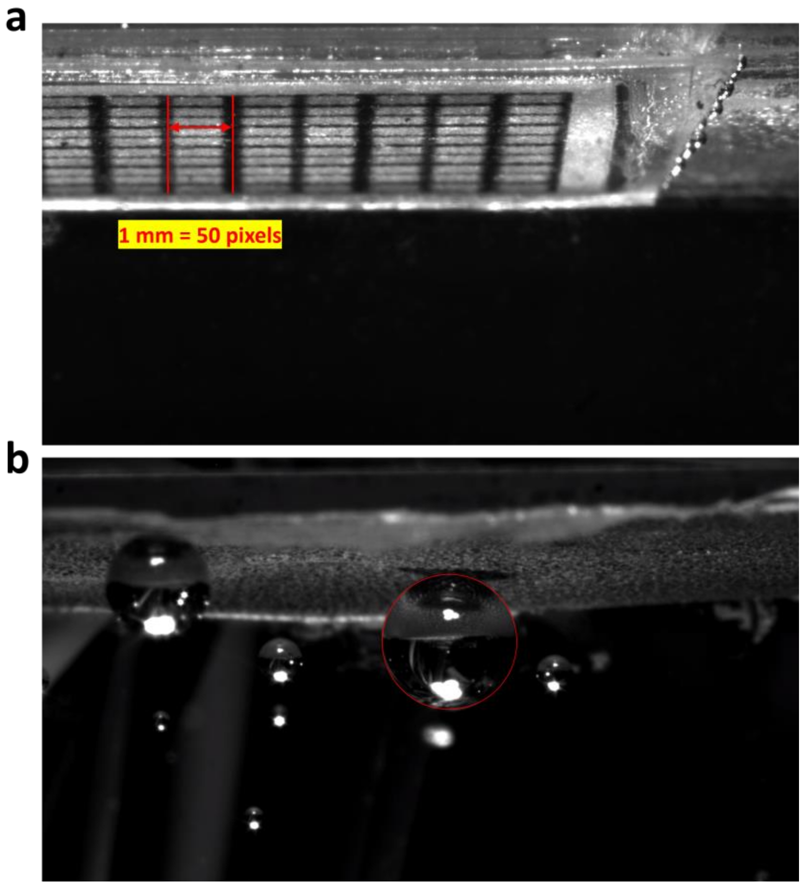

Supplementary Figure 10. (a) The optical image of the grid lines, which is used to convert the numbers of pixels obtained from the video to real sizes. (b) The image showing the precision achieved with this bubble fitting function in our MATLAB code.

Supplementary References:

1. Kestin, J., Sokolov, M. & Wakeham, W. A. Viscosity of liquid water in the range −8 °C to 150 °C. *J. Phys. Chem. Ref. Data* **7**, 941–948 (1978).
2. CHAPTER 2 - Flow Drilling: Underbalance Drilling with Liquid Single-Phase Systems, in *Underbalanced Drilling: Limits and Extremes* 39–108 (Gulf Publishing Company, 2012).
3. Zhang, Q. *et al.* Light-Guided Surface Plasmonic Bubble Movement via Contact Line De-Pinning by In-Situ Deposited Plasmonic Nanoparticle Heating. *ACS Appl. Mater. Interfaces* **11**, 48525–48532 (2019).
4. Zhang, Q. *et al.* Surface Bubble Growth in Plasmonic Nanoparticle Suspension. *ACS Appl. Mater. Interfaces* **12**, 26680–26687 (2020).
5. Zhang, Q., Li, R., Lee, E. & Luo, T. Optically Driven Gold Nanoparticles Seed Surface Bubble Nucleation in Plasmonic Suspension. *Nano Lett.* **21**, 5485–5492 (2021).
